# Evil’s Anatomy: Structural Correlates of Violent Behavior

**DOI:** 10.7759/cureus.102397

**Published:** 2026-01-27

**Authors:** Sergio Moreno-Jiménez, Mariana Olguín Gómez, Paola Mariana Quiñones Nájera, Axel Jared Carro Gallegos, Alejandro Salazar Pigeon, Daniel Ballesteros Herrera, Fabiola Flores-Vázquez, Marco Antonio Alegría Loyola

**Affiliations:** 1 Neurosurgery Department, Instituto Nacional de Neurología y Neurocirugía "Manuel Velasco Suárez", Mexico City, MEX; 2 Neurological Center, American British Cowdray Medical Center, Mexico, MEX; 3 Neurosurgery Department, Instituto Nacional de Neurologia y Neurocirugia "Manuel Velasco Suárez", Mexico City, MEX; 4 Cancer Center, American British Cowdray Medical Center, Mexico City, MEX; 5 Neurological Center, American British Cowdray Medical Center, Mexico City, MEX

**Keywords:** grey matter, neurobiology of evil, neuroimaging, prefrontal cortex, psychopathy, violent behavior

## Abstract

Evil has been approached from various disciplines, but its relationship with neuroanatomy remains an expanding field of study. This narrative review examines neuroscientific evidence on the brain structure of individuals who display *evil* traits, such as psychopathy and antisocial behavior. It analyzes how alterations in grey matter and neural connectivity may influence the manifestation of violent behavior. A literature search was conducted in PubMed, Web of Science, and Google Scholar, using ResearchRabbit to identify related articles. The keywords "anatomy of evil," "serial killers," "psychopathy," "brain structure and violent behavior," and "neurobiology of crime" were used. Studies on neuroimaging, neuroanatomy, and psychopathy published in peer-reviewed scientific journals were included. The findings indicate that alterations in grey matter volume in key structures, such as the prefrontal cortex (orbitofrontal, ventromedial, and dorsolateral), temporal lobe (amygdala and hippocampus), cingulate cortex, and insula, are associated with impulsivity, a lack of empathy, and aggressiveness. Likewise, abnormalities in white matter and corpus callosum volume and connectivity may contribute to deficits in emotional regulation and decision-making. Furthermore, individuals with a history of homicide exhibit significant neuroanatomical differences compared with other types of violent offenders. Although evidence suggests that the *predisposition to evil* may have a neurobiological basis, these factors are not deterministic. Environmental influences, such as childhood abuse and social deprivation, can modulate the impact of these brain alterations. Longitudinal studies and functional MRI are essential for distinguishing the causes from the consequences of the observed anatomical differences. In conclusion, there are neuroanatomical patterns that could be linked to violent and psychopathic behaviors, but their study still requires greater depth to establish causal relationships. Understanding the *neurobiology of evil* can provide valuable insights for criminology, forensic psychiatry, and the development of effective early intervention strategies.

## Introduction and background

The word evil comes from the Latin malĭtas, defined by the Royal Spanish Academy as "quality of evil" or "bad and unjust action" [1]. Evil can be studied from different disciplines, including psychology, ethics, anthropology, sociology, philosophy, and theology. Ponerology, derived from the Greek term panerós, is defined as the study of evil in its strict sense and aims to frame it as a human phenomenon, independent of its religious connotations. Under the premise that evil pertains to human nature [2], it is reasonable to theorize that its origins may be related to brain anatomy.

This work is a narrative review that integrates and analyzes the available neuroscientific evidence on the relationship between brain structure and *evil* behavior. Unlike previous studies that focus on isolated aspects of psychopathy or aggression, this review integrates key findings from neuroanatomy, neuroimaging, and neuropsychology to propose a broader conceptual framework for the neurobiological manifestations of *evil*. It is hypothesized that alterations in gray matter and in the functional connectivity between specific brain structures may predispose certain individuals to socially unacceptable behaviors, opening new perspectives in criminology, forensic psychiatry, and neuroethics.

Methodology

For the preparation of this narrative review, a literature search was conducted in the PubMed, Web of Science, and Google Scholar databases, complemented by the use of ResearchRabbit [3] to identify related articles and expand the network of relevant references. The keywords "anatomy of evil," "serial killers," "psychopathy," "brain structure and violent behavior," and "neurobiology of crime" were used. Studies were selected based on predefined inclusion and exclusion criteria. The articles included focused on macroscopic anatomy or used imaging techniques to compare groups of individuals with violent behavior confirmed through validated diagnostic tools. Studies that did not meet these methodological or population criteria were excluded. The detailed criteria are summarized in Table 1.

**Table 1:** Inclusion and exclusion criteria of the articles used in this narrative review.

Inclusion criteria	Exclusion criteria
Articles published between 1993 and 2024	Articles published before 1993 or after 2024
Focus on the study of macroscopic anatomy	Studies focused solely on microscopic anatomy or molecular biology
Studies including children, adolescents, and adults, regardless of their sex	Studies involving individuals with diagnosed mental illnesses (confirmed by diagnostic tools)
Use of imaging methods for comparisons	
Inclusion of participants with psychopathy, confirmed through diagnostic tools	

The concept of evil and its anatomical correlation

The *social brain* is one of the brain basis model of social interactions that incorporates many elements of various systems involved in socially significant aspects of human behavior. The *social brain* model comprises components of the reward system, the Theory of Mind (ToM) neural system and its various domains, affective and cognitive, as well as the systems supporting empathy. From the perspective of personality psychology, altogether with social intelligence, social behavior is affected by a set of non-pathological personality traits (non-pathological personalities), first described in the Theory of the Dark Triad by Paulhus and Williams in 2002. The triad includes: (1) narcissism - characterized by excessive self-esteem and arrogance; (2) psychopathy - characterized by a lack of empathy and remorse, as well as such phenotypic domains as disinhibition, arrogance and courage; (3) Machiavellianism - characterized by a tendency to manipulate and use others for manipulator’s purposes [4].

In 2018, psychologists Morten Moshagen, Benjamin Hilbig, and Ingo Zettler published "The Dark Core of Personality," in which they encompassed nine personality traits in what they called the "Dark Factor of Personality" or "D-factor," which is intrinsic to socially questionable behavior and can be interpreted as “evil”. These traits prioritize individual interests disregarding the potential harm to others. The “Dark Factor of Personality” includes selfishness, Machiavellianism, moral disengagement, narcissism, arrogance, psychopathy, sadism, self-interest, resentment, envy, and anger [5]. Dark traits are considered as specific manifestations of a general, basic dispositional behavioral tendency that in fact manifests the “Dark Factor of Personality”. It is believed that individuals with more prominent dark personality traits tend to be more prone to antisocial and dangerous behavior, which cannot but affect the nature of social interactions [4].

The literature discusses whether the Dark Triad traits are independent behavioral predictors or whether they should be combined within the framework of an integral assessment of the complex of traits (the dark core of personality). Despite the heterogeneity of the results obtained through the integrative approach that simultaneously considers all the subtests of the Dark Triad questionnaire, recent studies validate its efficiency. In this regard, we need to emphasize that we have not been able to find separate neurobiological studies that account for such integrity of the Dark Triad subtests indicators. As of now, the most widespread in the field are studies of the neuroanatomic organization of the individual Dark Triad traits based on specialized psychometric questionnaires [4].

Few studies have shown a positive correlation between the level of Machiavellianism (measured by a separate expanded specialized psychometric test MACH-IV) and the volume of some structures, such as subcortical nuclei, the prefrontal cortex, and the insula. Likewise, the meta-analysis by De Brito et al. demonstrated a gray matter volume decrease in the medial orbitofrontal and dorsolateral prefrontal cortex in psychopathy. Additionally, a number of studies have shown a positive correlation between the level of psychopathy (assessed using PCL-R questionnaires) and the volume of subcortical nuclei, in particular the putamen and caudate nuclei, although an inverse relationship is also reported. Meanwhile, the level of narcissism assessed using a neuropsychiatric Inventory questionnaire (NPI) positively correlated with the volume of a number of structures, including the medial prefrontal cortex, ventromedial prefrontal cortex, dorsolateral prefrontal cortex, orbitofrontal cortex, middle anterior cingulate cortex, and the insula. Notably, in the case of the pathological variant - the narcissistic personality disorder - the degree of manifestation of the disease negatively correlated with the volume of the medial, prefrontal and dorsolateral cortex [4].

Myznikov et al. [4] conducted a morphometric study of dark personality traits using the general Dark Triad questionnaire (Dirty Dozen Dark Triad, DDDT) and the k-means data clustering algorithm, which provides an adequate approach to the multifactorial structure of the Dark Triad and allows identification of groups based on a data-driven approach. Furthermore, they performed a morphometric analysis on the resulting groups to assess differences in gray matter volumes across Dark Triad profiles. Clusters were designated as *mid-to-high DT level* and *low-to-mid DT level*. The voxel-based morphometry (VBM) analysis revealed a decrease in gray matter volume in the mid-to-high DT level group in several regions, including the prefrontal cortex (medial and lateral orbitofrontal cortices), basal ganglia (bilateral nucleus accumbens, putamen, and left caudate nucleus), middle cingulate cortex, and right postcentral gyrus. These results suggest a possible relationship between the prominence of Dark Triad traits and the volume of structures associated with socio-emotional functions, such as empathy and emotional regulation.

The vast majority of studies [6-10] on violent behaviors highlight that adolescence is a critical period of biological, cognitive, and neural changes, which may be associated with daring, irresponsible, and criminal behavior. This period represents the basis for two variants of behavioral disorders: a persistent antisocial trajectory of early onset, with a prevalence of less than 10%, and antisocial behavior limited to adolescence, with a prevalence greater than 25%. Current evidence suggests that individuals with a persistent antisocial trajectory have neuropsychological vulnerabilities that, together with various environmental factors, impair the development of social skills and, therefore, maintain them on the antisocial spectrum [11].

The most frequent finding in individuals with problematic behavior is a reduction in gray matter volume across various brain areas, particularly those involved in cognition and emotion regulation. However, Pujol et al. [12] note that, although there is vast information on variations in gray matter volume, these changes are not usually macroscopically evident and are likely inversely related to increases in white matter, especially in the prefrontal cortex and the corpus callosum.

Sajous-Turner et al. [13] described one of the most significant challenges in understanding structural differences in individuals who fit within the spectrum of *evil*. They noted that in most studies involving homicide offenders who were found not guilty by reason of insanity, the presence of an underlying psychiatric condition makes it difficult to distinguish homicidal behavior from the manifestations of the disorder.

Psychopaths understand moral precepts and social rules, yet they remain indifferent to both the rules and the consequences of not following them. When faced with ethical dilemmas, they do not hesitate to cause harm if it helps them achieve their goals [14]. This study aims to provide the most accurate and up-to-date information on the brain anatomy of individuals who fit into the category of “evil”. This concept is subjective and multifaceted, which limits the study to some of the many faces of this human condition. We also adopt a concept introduced by Sajous-Turner et al. [13], which suggests that the anatomical relationships described to date are not determinants of psychopathic or homicidal behaviors, but rather guide research toward studying aberrant neural connectivity within brain systems involved in cognition and emotional regulation. Pujol et al. [12] also addressed this theory, noting a dynamic evolution of gray matter as individuals age, supported by studies indicating that children under 12 years of age with psychopathic traits exhibit increased gray matter volume. This finding contrasts with evidence in adolescents (17 years or older) with psychopathic traits, who show decreased gray matter volume. Additional studies consistent with this theory address the influence of childhood abuse and adverse life events on gray matter volume in psychopathic individuals [15-18]. 

## Review

Gray matter correlates

Frontal Lobe and Prefrontal Cortex

**Figure 1:**
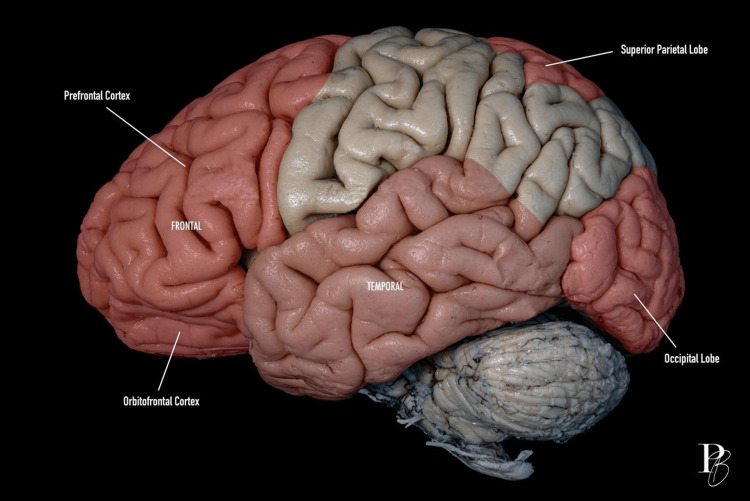
Lateral view of the cerebral lobes and cortical regions. Lateral view of the human brain showing the main lobes and cortical regions. The frontal lobe is highlighted in red, including the prefrontal and orbitofrontal cortex. The parietal lobe, shown in beige, includes the superior parietal lobe. The temporal lobe is depicted in pink. The occipital lobe is also in red. The cerebellum is visible at the bottom. The photograph was taken by Dr. Daniel Ballesteros-Herrera, co-author of the work.

Figure 1 shows a lateral view of cerebral lobes and cortical regions. 

The frontal lobe, particularly the prefrontal cortex (PFC), is involved in executive functions, which are cognitive processes that allow goal-directed behavior, including problem-solving. Four domains often discussed in relation to executive functions include volition, planning, intention, and execution. Each function requires the coordination of behavior and cognition, and the brain areas involved in these processes are frequently studied in behavioral disorders [19]. Early studies on the PFC highlighted its role in personality and behavior, arising from the accident suffered by worker Phineas Gage in 1848, in which a metal rod pierced the frontal lobe of his brain. Gage became impulsive and uninhibited, with reduced empathy and impaired decision-making. Subsequent research has shown that frontal lobe damage is associated with poor impulse control, aggressiveness, socially inappropriate behaviors (including disinhibited sexual behavior), impaired judgment, and poor understanding of long-term consequences [20].

Orbitofrontal Region

The orbitofrontal cortex (OFC), a ventral region of the PFC, is highly involved in decision-making, behavioral regulation, emotional processing, and social interaction. It maintains strong reciprocal connections with the amygdala, other limbic structures, and additional prefrontal regions. Neuroimaging studies indicate that the OFC is engaged during the regulation and suppression of aggressive and violent impulses; therefore, damage to this region can disrupt these regulatory mechanisms [21]. Structural neuroimaging research has also reported grey matter volume alterations in the OFC of violent offenders [11,13,16]. Nevertheless, these findings must be interpreted cautiously, since many studies present limitations or potential confounders, such as small sample sizes [13-14,16,22-26], comorbid psychiatric histories [22,27], findings applying only to male population [23,26-27], substance-use histories [14,28-29], reliance on cross-sectional designs [22-23,26], or the inability to examine brain responses in other aggressive populations [13,22].

Huebner et al. [27] reported that 23 adolescents with conduct disorder had a 6% decrease in the amount of grey matter in the left OFC. Similarly, multiple articles published over the last 20 years have linked grey matter decrease with emotional callousness and impulsive behavior [24-26,30]. In 2011, the "Vietnam Head Injury Study and 40 years of Brain Injury Research" yielded the results of an analysis carried out over forty years of a cohort of 1,221 war veterans with penetrating brain injuries, showing that up to 14% of them who had frontal lobe injuries were involved in fights or damage to private property, compared to 4% without injuries. When subjected to imaging studies to identify the location of the lesions, a significant association between damage to the OFC and increased aggressiveness was found [31]. Three years later, Cope et al. [30] compared the brain structure of 3 groups: adolescents with conduct disorder who committed homicide, adolescents with conduct disorder without committing the crime above, and healthy adolescents. This study found that young people who committed murder had a 5% smaller total brain volume compared to those who had a conduct disorder but did not commit the crime, with marked differences in the grey matter of the medial orbitofrontal cortex (mOFC). The authors conclude that although the findings are relevant, they should be interpreted cautiously as it is unknown whether this reduction in volume developed over time or whether the subjects were born with it, and it is unlikely that it is feasible to subject all adolescents at risk of developing a conduct disorder to serial functional imaging studies.

Medial, Lateral, and Ventral Regions

The PFC can be functionally and anatomically divided into medial, lateral and ventral regions. The medial prefrontal cortex (mPFC) comprises the dorsomedial (dmPFC) and ventromedial (vmPFC) areas, both of which are present across mammals. The lateral prefrontal cortex (lPFC) includes the dorsolateral (dlPFC) and ventrolateral (vlPFC) subdivisions, which show greater evolutionary expansion in primates. The ventral prefrontal cortex (vPFC) refers broadly to ventral aspects of the PFC, including regions involved in emotion regulation and value-based decision-making [32]. Additionally, the mPFC components display different functions: the dmPFC responds more strongly to information about others, whereas the vmPFC is more engaged in self-referential processing [33].

The vmPFC guides behavior by processing reward and punishment related stimuli, inhibiting actions driven by inappropriate motivations, and regulating aggressive behavior [19]. It is involved in social and affective functions that are essential for behaviors requiring consideration of shared or common interests. This region also contributes to the processing of social norms and cultural values, the generation of empathic responses, risk estimation, moral judgment, trial-and-error learning, and the recognition of emotional facial expressions. The maturation of moral behavior is closely linked to the proper development of the ventromedial region [20]. Boes et al. [34] reported a case in which malformations in this area impaired social development and ultimately lead to antisocial behavior. This is not surprising, as several studies have identified structural abnormalities in this region in men with antisocial personality disorder (ASPD), type II alcoholism, a history of substance use, and in patients with psychiatric disorders associated with impulsivity and aggression. More recently, both structural and functional studies have identified abnormalities in vmPFC-amygdala connectivity in psychopaths and in adolescents with behavioral disorders [19].

Similarly, the dlPFC is associated with impulse management and mental functions, including risk-benefit assessments, problem-solving, and cognitive control. This region interacts closely with the ventrolateral region in moral decision-making processes, as the former is responsible for inhibiting intense emotional reactions, and the latter is activated in self-control. Functional and anatomical abnormalities in the dorsolateral region have been associated with the development of impulsive behavior, deficits in aggression control, and altered reward processing, which may lead affected individuals to experience gratification when engaging in criminal or violent actions [20]. Baumgartner et al. [35] reported that subjects with greater grey matter volume in the dmPFC, as determined through quantitative morphometric magnetic resonance imaging (MRI) analyses, tend to act more fairly and are less easily influenced. This finding is relevant because compromised impartiality and a tendency to act unfairly can contribute to antisocial and aggressive behaviors.

In 2019, Sajous-Turner et al. [13] published the study "Aberrant Brain Gray Matter in Murderers," in which they sought to address a key methodological limitation in research comparing violent and non-violent individuals: under such broad classifications, it is not possible to determine whether brain differences vary according to the degree of violence. Using MRI data approved by the National Institutes of Health, the authors compared brain structure across three groups: (1) a homicidal group (individuals who confirmed having committed murder); (2) a violent but non-homicidal group (individuals who committed offenses such as aggravated robbery, crimes involving physical contact with the victim, domestic violence, kidnapping, or arson); and (3) a minimally violent group (individuals who committed offenses such as robbery, driving under the influence, drug or weapon possession or trafficking, arson without victims, child abuse, voyeurism, possession of child pornography, kidnapping without direct contact, or vandalism). The findings revealed that individuals in the homicidal group showed distinct bilateral grey matter alterations compared with both the violent but non-homicidal and minimally violent groups, particularly in the vmPFC, vlPFC, dmPFC, dlPFC, and OFC. In contrast, no significant structural differences were observed between the violent but non-homicidal and minimally violent groups. It is important to mention that although the analysis of variance did not reveal statistically significant differences in total brain volume across groups (*F* = 2.730, *P* = 0.0666), the results suggest a trend toward higher values in the minimally violent group compared to the homicidal and violent but non-homicidal groups. While this finding does not reach the conventional threshold for significance (*P* < 0.05), the proximity of the *P*-value may indicate an underlying effect that was not detected due to sample size limitations, as reported by the authors. From a neurobiological perspective, this trend could be relevant, as greater total brain volume might be related to structural or functional cortical differences associated with impulse control and emotional regulation.

Parietal Lobe

Superior parietal cortex: The parietal lobe lies posterior to the central sulcus and superior to the lateral sulcus, extending posteriorly until it reaches the parieto-occipital sulcus. The lateral surface of the parietal lobe is divided by two sulci into three main regions. The postcentral sulcus runs parallel to the central sulcus, and between them lies the postcentral gyrus. The intraparietal sulcus extends posteriorly from the middle portion of the postcentral sulcus. Above the intraparietal sulcus is the superior parietal lobe (SPL), and below it the inferior parietal lobe (IPL) [36].

The SPL, corresponding primarily to Brodmann area 7, is responsible for multisensory and visuomotor integration, and maintains reciprocal connections with the PFC [36]. Descriptions of potential anomalies in this region date back to 1997, when British psychologist Adrian Raine, author of multiple studies on violence and psychopathy, reported reduced brain activity in the prefrontal, temporal and parietal cortices of individuals who had committed homicide [37]. Similar findings were later described by Boccardi in 2011 [29] and Bertsch in 2013 [24]. Sajous-Turner et al. [13] identified decreases in grey matter beginning in the precuneus (Brodmann area 7), extending across the SPL (Brodmann area 7), the IPL (Brodmann area 40), and the postcentral gyrus (Brodmann areas 1-3).

Temporal Lobe

The temporal lobe functions include memory retention and storage, the organization of sensory stimulus input, language production, visual perception, and emotional response [19]. The anterior temporal cortex (aTC) and the PFC are responsible for evaluating and integrating information related to social interactions, which is known as ToM and empathy. These skills are essential for effective social functioning and appropriate adaptive behavior, so if this region is affected, it can lead to an aggressive response toward others [13].

The study of the association between the temporal lobe and violent and antisocial behaviors began in 1949 with electroencephalogram studies in individuals who had committed homicide, where researchers detected abnormal electrical activity. Since 2000, multiple studies have examined potential structural differences in the brains of violent individuals and/or those with antisocial personality traits, reporting up to a 20% bilateral reduction in grey matter volume in this region compared with healthy controls [19].

Cope et al. [30] made a comparison between brain volumes in incarcerated male adolescents who committed homicide and incarcerated offenders who did not. This analysis is valuable because the structural differences found, such as lower total brain volume (including both lower total grey and white matter) and reduced grey matter volumes in large bilateral temporal lobe clusters, hippocampus, posterior insula, superior temporal gyrus, middle temporal gyrus, parahippocampal gyrus, fusiform gyrus, and inferior temporal gyrus, are not attributed to the difference in age, intelligence quotient (IQ), socioeconomic status, score on the Hare Psychopathy Checklist in its adolescent version, emotional callus, impulsivity, brain injury, mental illness, number of crimes, or substance use, and although the authors recognize that committing a homicide is a behavior and not a disorder, there must be a factor that determines this action.

In "Aberrant Brain Gray Matter in Murderers," structural differences were found in the aTC of individuals who committed homicide [13], a result consistent with those obtained by Cope et al [30]. The fact that both studies adopted a methodology in which the characteristics of the participants and their actions are used as covariates makes it unlikely that the differences found could be due to factors other than the ones analyzed. The conclusion of "Aberrant Brain Gray Matter in Murderers" is essential since it supports the theory that there is a determining factor for homicidal action, as it does not find significant differences in the brain structure of violent but non-homicidal individuals and minimally violent individuals.

Occipital Lobe

The occipital lobe contains the primary visual cortex (Brodmann area 17), responsible for the reception of visual input, as well as the secondary visual cortices (Brodmann areas 18 and 19), which are involved in the processing, recognition, and interpretation of visual information [36].

Bertsch et al. [24] published one of the first papers to focus on subgroups of violent people. The authors main objective was to homogenize the evidence that existed up to that point of grey matter volume variations in antisocial offenders with either borderline personality disorder (ASPD-BPD) or high psychopathic traits (ASPD-PP), compared to healthy controls. This comparison was made through MRI, where the volumetric differences of the regions of interest were analyzed by VBM in combination with high-dimensional image deformation.

In addition to finding differences in previously studied regions, such as the PFC and the temporal lobe, this study reports a decrease in the volume of gray matter in the left occipital cortex in ASPD-BPD and ASPD-PP compared to controls; however, the study of this region was not deepened due to the lack of hypotheses that correlate it with these features [24].

Limbic System

Cingulate cortex: The cingulate cortex is located on the medial aspect of the cerebral cortex, including the cingulate gyrus, which follows the contour of the corpus callosum, and the grey matter that lines the upper and lower edges of the cingulate sulcus [38]. It comprises abundant spindle neurons, which have only been found in humans and animals with remarkable intelligence, such as apes, cetaceans, and elephants [21]. Functionally, it is divided into four regions: the anterior cingulate cortex (ACC), the medial cingulate cortex (MCC), the posterior cingulate cortex (PCC), and the retrosplenial cortex (RSC) [38].

The ACC is divided into dorsal and ventral segments, and it is also interconnected with the PFC, parietal cortex, frontal eye fields, amygdala, hippocampus, hypothalamus, the anterior insula, and the nucleus accumbens [21]. Its functions include monitoring both internally generated information and environmental stimuli, directing attention to the most relevant inputs at any given time [19]. The ACC is also responsible for detecting errors, integrating incongruous situations (e.g., the Stroop effect), perseverance, empathy, and completing delayed tasks. Ultimately, it is responsible for assessing stimuli that induce fear and anxiety, thereby modulating the autonomic response and fear-related behaviors [21]. It is essential to mention that cognitive information in this region is processed separately from emotional information [20].

Hornak et al. [39] described that patients with unilateral lesions in the ventral ACC had difficulty identifying voices and facial expressions, as well as changes in behavior and emotional processing. In 2009, Yang and Raine published a meta-analysis of 43 independent studies, concluding that antisocial behavior arises from defects primarily concentrated in the right cerebral hemisphere. These results complemented the perspective initially provided by Hornak et al. in 2003. In the same study, the authors mention that specific damage to the OFC and the ACC can indirectly worsen attention, as an injury to these areas can misdirect stimuli by playing an essential role in emotional processing [40]. In the same way, they supported the data reported by Kosson in 1996 in "Psychopathy and Dual-Task Performance Under Focusing Conditions," who mentioned that psychopathic individuals have attention problems due to difficulties in processing information in the left hemisphere and in shifting attention to the right hemisphere [41].

Regarding the amount of gray matter in this region, Cope et al. [30] pointed out a decrease in the anterior portion of the cingulate gyrus. This finding was supported in 2019 by Sajous-Turner et al. [13], who specified that the reduction was restricted to the dorsal ACC, the MCC, and the PCC.

**Figure 2:**
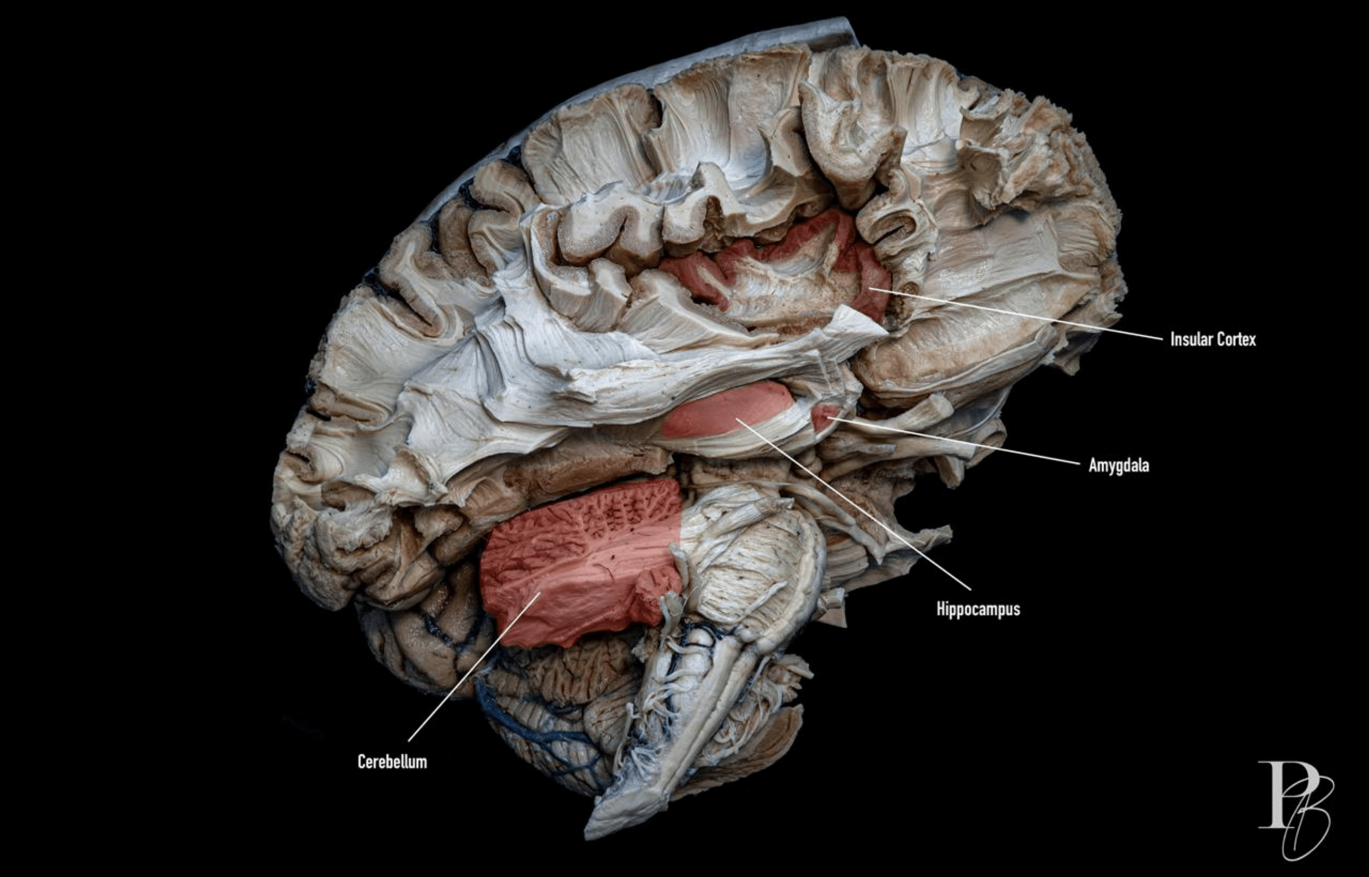
Medial dissection of the limbic system and insular cortex. This view provides insight into the anatomical relationships of the limbic system and brainstem structures. The insular cortex, located deep within the lateral sulcus, is observed. The amygdala is highlighted in red, and the hippocampus lies just below it. The cerebellum is visible at the base of the brain. The photograph was taken by Dr. Daniel Ballesteros-Herrera, co-author of the work.

Insula: Figure 2 shows a medial dissection of the limbic system and insular cortex. 

The insula is an area of the cortex folded deep within the lateral sulcus, from which it forms its floor. It can only be examined when the lips of the lateral groove are widely separated. The insula is considered a structure of the limbic system that underwent early embryological development and has preserved its functions throughout the maturation process of the central nervous system. Like the cingulate cortex, the insula contains many spindle neurons, which is why it is also credited with integrating and processing contradictory information [21]. It is divided into anterior and posterior regions, the anterior region receives information from the thalamus and sends it to the amygdala, while the posterior region is connected to the secondary somatosensory cortex and the thalamus [36].

Regarding the anterior region, Krämer’ et al.'s [42] and Veit et al.’s [43] findings indicated that, in conjunction with the cingulate cortex, the ventral and dorsomedial PFC, the hypothalamus and the striatum, this region is involved in the processing of stimuli perceived as aggressive. On the other hand, Craig et al. [44] and Farrer et al. [45] reported that the posterior insula is involved in action capacity, interoception, and the sense of identity. Furthermore, Xue et al. [46] suggested that the insula as a whole represents homeostatic states associated with the experience of risk; consequently, individuals with impaired risk monitoring may be more prone to engaging in homicidal behavior.

In terms of the relationship between brain volume and homicidal behavior, Cope et al. [30] reported a significant reduction in posterior insula volume in adolescents. This finding aligns with the results of Sajous-Turner et al. [13], whose study analyzed an adult population.

Amygdala: The amygdala complex is located on one side in front and the other above the tip of the inferior horn of the lateral ventricle. It fuses with the tip of the tail of the caudate nucleus, which has passed forward on the roof of the inferior horn of the lateral ventricle. The stria terminalis emerges from its posterior aspect [36]. It is divided into three main components: the basolateral nucleus, the central nucleus, and the corticomedial nucleus. The basolateral nucleus, in turn, contains the basal, lateral, and accessory basal nuclei, while the corticomedial nucleus contains the cortical and medial nuclei. The basolateral nucleus is crucial in assigning meaning to stimuli, integrating the emotional response to memory and augmenting the autonomic nervous system's response. Sex-related structural differences have been described: men tend to show a larger right amygdala, whereas women show a larger left one. Functionally, the right amygdala is linked to fear and sadness responses, while the left is associated with consciousness and language processing [21].

The amygdala regulates social interactions by processing and responding to emotional stimuli and is also involved in the recognition of emotions through facial expressions [21,47]. For humans, facial expressions represent a means of nonverbal communication, providing an emotional component to social interactions. The neural circuit between the amygdala and the PFC is thought to be responsible for interpreting emotions coming from angry faces [19]. This is relevant because defects in the connectivity of this circuit are thought to impair information processing (e.g., misinterpreting a neutral facial expression as an aggressive one), which may ultimately lead to violent behavior. One of the conditions studied in relation to this phenomenon is intermittent explosive disorder (IED), a chronic condition characterized by repetitive and unplanned outbursts of anger, disproportionate in intensity to the triggering event, which can significantly affect the individual's interpersonal relationships [22].

For years, the amygdala has been considered a structure of interest in individuals with disorders associated with violent behavior. The leading hypotheses suggest that defects in the amygdala or other parts of the limbic system impair the interpretation of danger signals, resulting in excessive aggressiveness [48].

Sterzer et al. [47] aimed to determine, using VBM, whether there were structural differences in the regions associated with emotional processing in adolescents with conduct disorders. The authors found a significant reduction in gray matter in the bilateral anterior insular cortex and in the left amygdala; the latter being primarily associated with attention problems and aggressive behavior. Attention problems support the theory linking the amygdala to the recognition of emotions through facial expressions; therefore, reduced gray matter volume in this area is associated with difficulties in social interaction.

A study by Huebner et al. [27] analyzed abnormalities in gray matter volume in a sample of 23 adolescents with conduct disorder, aged 12-17, using MRI and VBM. In this study, the results concluded that, compared to healthy controls, young people with conduct disorder had an overall decrease of 6% in gray matter, especially in the left orbitofrontal region, both temporal lobes, the left amygdala, and the hippocampus.

These findings were confirmed again by Cope et al. [30] in 2014, who used high-resolution T1-weighted structural MRI scans and VBM to report a bilateral reduction in amygdala gray matter among individuals who had committed homicide.

During that same year, Pardini and Raine published a longitudinal study aimed at determining whether men with reduced amygdala gray matter volume had a history of childhood violence and psychopathic traits, and whether this characteristic could be considered a predictor of future violent behavior. The authors found that reduced volume of the right and left amygdala was associated with aggressive behavior and premeditated aggression. More specifically, reduced gray matter volume in the left amygdala was linked to higher levels of impulsive-affective-reactive aggression (IAR), as well as childhood callousness. In contrast, reduced gray matter volume in the right amygdala was associated with proactive aggression and psychopathic traits during adolescence. They also reported that bilateral reductions in gray matter within this region were closely associated with an increased risk of committing violent acts in the future. The authors concluded that this was the first study to demonstrate such an association and that amygdala volume constitutes a key factor in violent and psychopathic behaviors during childhood [18].

Yoder et al. [49] aimed to investigate the relationship between psychopathic traits and the amygdala’s response to violence, using probabilistic tractography to classify the amygdala subnuclei based on their anatomical projections within a group of 43 patients. While unique connectivity was observed between the amygdala and the right medial occipital gyrus, each subnucleus displayed distinct connectivity patterns. The basolateral nucleus exhibited neuronal coupling with the anterior cingulate and prefrontal regions, whereas the central nucleus showed strong connectivity with the brainstem but limited connections to the superior parietal and precentral gyri. Furthermore, high impulsivity scores on the Psychopathic Personality Inventory (PPI-R) were associated with reduced neuronal coupling between the basolateral nucleus and its ventrolateral region in the PFC, and scores related to fearless dominance were associated with increased neuronal coupling between the central nucleus and the posterior insular region. Overall, this study supports the notion that the amygdala does not function as a unitary structure, but rather as a complex assembly whose subcomponents contribute differently to violent behavior [21].

Hippocampus and Parahippocampal Cortex

Evidence obtained through electrophysiology, functional neuroimaging, and injury studies classifies the paralimbic cortex and limbic system as dysfunctional in psychopathic individuals. The structures that share cytoarchitecture include the temporal lobes, the ACC, the PCC, the OFC, the parahippocampal region, the amygdala, and the hippocampus, suggesting a common neurodevelopmental origin [50].

The hippocampal formation comprises the hippocampus, the dentate gyrus, and the parahippocampal gyrus. The hippocampus is a curved ridge of gray matter extending along the floor of the inferior horn of the lateral ventricle, with its anterior portion forming the hippocampal head. The dentate gyrus is a narrow, dentate band of gray matter located between the hippocampal fimbria and the parahippocampal gyrus. Anteriorly, the dentate gyrus continues at the uncus. The parahippocampal gyrus lies between the hippocampal fissure and the collateral sulcus, extending medially along the temporal lobe toward the hippocampus [36].

The hippocampus plays a fundamental role in learning and memory, specifically in consolidating memories related to facts and events. Together with the parahippocampal gyrus, it is thought to be involved in regulating aggression and fear conditioning, as well as in modulating information related to impulse control and moral reasoning [19].

Addressing brain volume in this region, Huebner et al. [27] found reduced hippocampal gray matter in a sample of 23 adolescents with conduct disorder aged 12-17 years. A year later, De Brito et al. [23] described increases in the volume and concentration of gray matter in a sample of 23 boys with callous-unemotional traits, aged 10-13 years. The increases were observed in the left posterior hippocampus, the posteromedial OFC, the dorsal ACC, the superior parietal lobe, the bilateral superior temporal gyri, the postcentral gyrus, the superior frontal gyrus, and the cuneus [23]. These dual findings led to the theory proposed by Pujol et al. [12], which suggests dynamic changes in gray matter as individuals age.

In 2013, Ermer and Cope found a relationship between psychopathic traits in incarcerated adolescents housed in a maximum-security facility and reduced gray matter volume in several brain regions, such as the OFC, the parahippocampal cortex within both temporal lobes, and the PCC. Psychopathic traits were assessed using the Hare Psychopathy Checklist in its adolescent version, and the volume per region was determined through VBM [50]. A year later, Cope et al. [30] explored this finding in depth and described a significant reduction in bilateral hippocampal gray matter in adolescents who committed homicide, compared with healthy controls.

Thalamus and Hypothalamus

The thalamus is a large ovoid mass of gray matter that forms most of the diencephalon, located near the center of both cerebral hemispheres, close to the third ventricle and the basal nuclei [36]. It comprises 20 nuclei, functioning as a relay center, directing and exchanging sensory information between the different brain areas [19].

Evidence implicating specific thalamic nuclei in violent behavior is illustrated by the case report of Muneoka et al. [51], who described a 91-year-old woman with Alzheimer’s disease associated with delusions and hostility toward her family, frequently appearing angry and exhibiting resistance and violence toward caregivers. At the age of 95, she was diagnosed with an acute brain infarction that caused a restricted lesion in the right anterior thalamic nucleus (ANT); following this event, the patient’s emotional and behavioral aggressiveness disappeared, and she “talked gently with the care staff and received assistance without refusal or aggression.” In an assessment using the Behavioral Pathology in Alzheimer’s Disease Rating Scale, the patient’s scores for threat, violence, anxiety, and fear decreased by at least 2 points compared to pre-infarction levels. The ANT connects the temporal lobe to the PFC and is a known component of Papez’s circuit. Damage to the ANT is typically associated with memory and motor performance deficits; therefore, in the present case, the improvement in emotional behavior following an ANT infarction represents a paradoxical outcome. In summary, the authors conclude that the ANT may serve as a potential target for treating emotional disturbances in dementia involving severe temporal lobe pathology.

Another thalamic nucleus that might be involved in aggressive behavior is the ventral posterior nucleus (VPN), as reported by Yan et al. [52]. The authors described the case of a nine-year-old girl who underwent centromedian (CM) nucleus deep brain stimulation (DBS) implantation for drug-resistant epilepsy and developed unexpected and reversible post-stimulation aggressive behavior. With connectomic analyses, it was demonstrated that stimulation-induced aggressiveness engaged PFC-bound white matter fiber tracts as well as the salient neural circuitry involving prefrontal thalamocortical projections. This last area was located in the left thalamus, overlapping the VPN and encroaching on the posterior aspect of the posterior limb of the internal capsule. The authors acknowledge that the study’s reliance on a single patient limits the ability to precisely define the area responsible for the symptoms and to pinpoint the relevant fibers; however, the involvement of prefrontal thalamocortical circuitry in aggression following DBS is supported by literature implicating the PFC in aggressive or violent behaviors [52].

On the other hand, the hypothalamus is the part of the diencephalon that extends from the optic chiasm region to the caudal border of the mammillary bodies in the center of the limbic system. It is located below the thalamus and forms both the floor and the lower part of the lateral walls of the third ventricle. Despite its small size, it is essential for life, as it regulates the autonomic nervous and endocrine systems, undertaking appropriate control responses by integrating the nervous and chemical data it receives [36].

Veit et al. [43] studied brain activation and behavior of psychopathic individuals using a modified version of Taylor's aggression paradigm, analyzing the neural and behavioral responses of ten psychopathic criminals during the punishment phase. The study demonstrated that reactive aggression is mediated by the medial amygdala, the medial hypothalamus, and the dorsal region of the periaqueductal gray matter. Results showed that punishment elicited increased neuronal activation in the hypothalamus, the lateral PFC, the PCC, and the amygdala. Furthermore, a direct relationship was observed between physical aggression and hypothalamic activation. In conclusion, these findings highlight the role of the hypothalamus as a mediator of reactive aggression, which appears to be associated with the antisocial dimension of psychopathy rather than the affective or interpersonal dimensions.

Basal Nuclei: Caudate, Striatum, and Nucleus Accumbens

**Figure 3:**
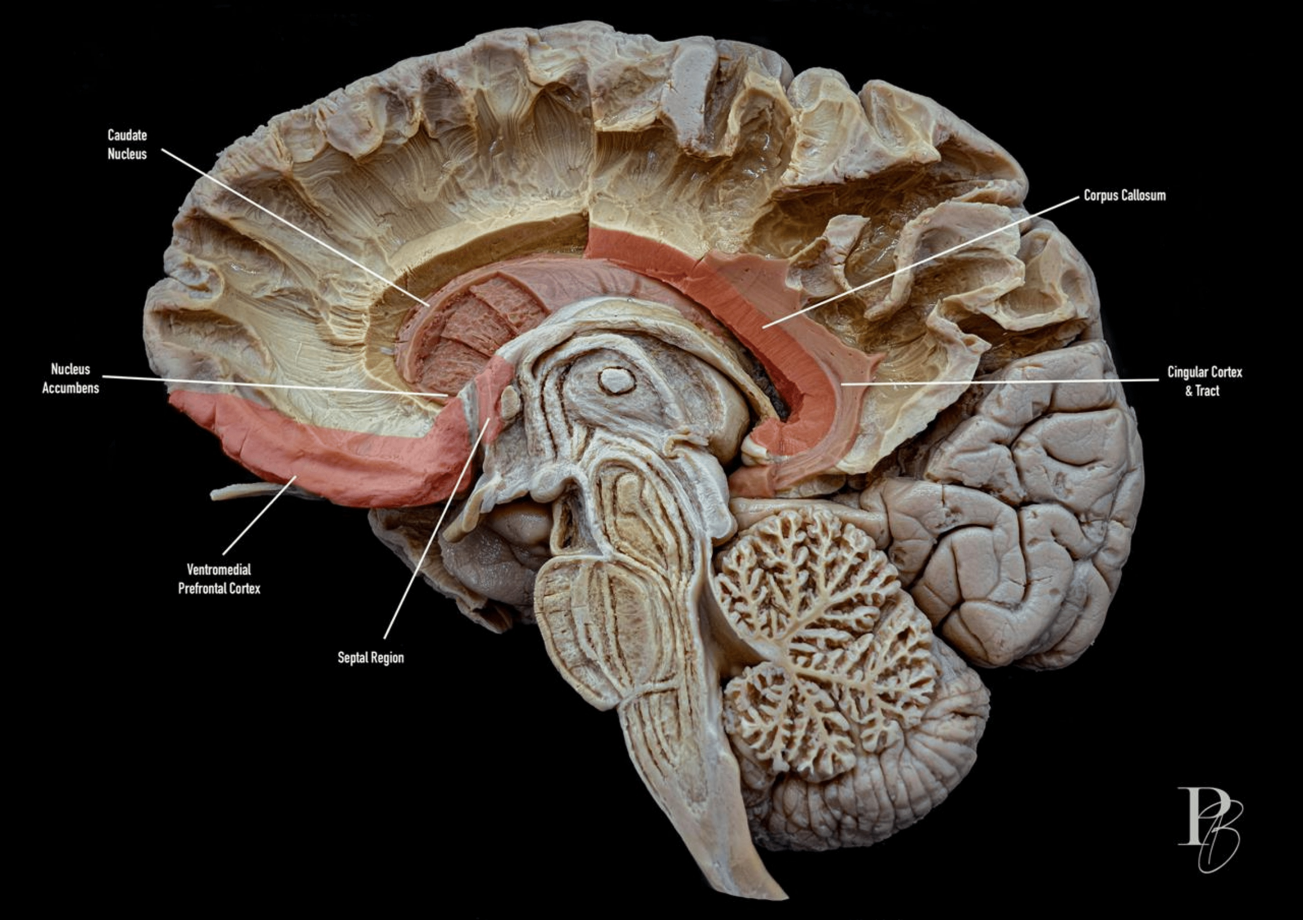
Midsagittal view of limbic structures and interhemispheric connections. Midsagittal section of the human brain highlighting key components of the limbic system and associated structures. The caudate nucleus and nucleus accumbens are shown in red, and the ventromedial prefrontal cortex lies anteriorly. The septal region is indicated, while the corpus callosum, located just above the cingulate cortex and tract, connects the two cerebral hemispheres. The cerebellum is visible inferiorly. The photograph was taken by Dr. Daniel Ballesteros-Herrera, co-author of the work.

Figure 3 shows a midsagittal view of limbic structures and interhemispheric connections.

The basal nuclei comprise a circuit of seven nuclei that are involved in movement planning, reward-based learning, and action selection [21].

The caudate nucleus is a large C-shaped mass of gray matter closely related to the lateral ventricle and located lateral to the thalamus. Its structure is divided into three elements: head, body, and tail. The head is large and rounded, and inferiorly it blends with the putamen of the lenticular nucleus. Immediately above this junction are bands of gray matter that course through the internal capsule, giving the region its characteristic striated appearance from which the term “striatum” originates. The body contributes to the floor of the lateral ventricle. The tail follows the curvature of the lateral ventricle, arching posteroinferiorly and then anteriorly along the roof of the inferior horn, where it terminates in the amygdaloid complex. Together, the caudate nucleus and putamen are often referred to as the *neostriatum* or simply the *striatum* [36].

The striatum is lateral to the thalamus and is divided almost entirely by the internal capsule, which passes between the caudate nucleus and the lenticular nucleus. It receives afferent information from most of the cerebral cortex, thalamus, subthalamus, and brainstem, including the substantia nigra [36].

The nucleus accumbens is a component of the basal nuclei that, together with the olfactory tubercle, forms the ventral portion of the striatum. It has two major subdivisions, the core and the shell, which contain neurons with distinct morphological and functional characteristics. Despite these differences, both subdivisions participate in behaviors related to aversion, fear, impulsivity, motivation, reward-seeking, and reinforcement learning. The nucleus accumbens receives afferent input from the basolateral nucleus of the amygdala, the PFC, and the ventral tegmental area, and sends efferent projections to other basal nuclei and, indirectly, to the OFC, the thalamus, the striatum, the substantia nigra, and the reticular formation. Because it receives substantial input from the amygdala and conveys information to the OFC, it is thought to play a key role in the regulation of goal-directed and emotional behavior [21].

Most studies that aim to measure aggressive behavior in humans examine neural responses during modified versions of Taylor's aggression paradigm [53,54]. Krämer et al. [42] investigated the affective and cognitive processes of healthy individuals throughout each phase of the experiment to determine whether reactive aggression is associated with specific brain structures. Their results showed that greater provocation by the opponent led participants to select more severe punishments. To explore whether administering punishment was intrinsically pleasurable, the authors analyzed participants’ choices when deciding on the punishment for the opponent who delivered the highest level of provocation. This comparison revealed that, during decision-making, activation occurred in the dorsal striatum (specifically, the body of the caudate nucleus), which is involved in reward processing, as well as in the dorsal ACC. This pattern suggests that choosing a severe punishment was experienced as gratifying. However, further analysis indicated that the participants’ sense of gratification was linked to the belief that severe punishment would have an educational or corrective effect on the opponent, rather than deriving satisfaction from the opponent’s suffering itself.

In 2010, Glenn and Raine published a case-control study comparing striatal volume in 22 individuals with psychopathy and 22 healthy controls using MRI. The results showed a 9.6% increase in the total striatal volume of psychopathic individuals. They also examined the relationship between the volume of specific striatal subdivisions and psychopathic traits, finding that enlargement of the caudate body was associated with interpersonal and affective traits, whereas enlargement of the caudate head was associated with impulsive and reward-seeking traits. These findings align with the evidence showing that psychopathic individuals exhibit a heightened drive for stimulation, maladaptive decision-making patterns, and strong performance in reward-based tasks. Likewise, striatal activity has been linked to individual differences in impulsivity, reflecting a preference for immediate rewards over long-term ones. The relevance of this study lies in its support for the theory that increased sensitivity to reward may contribute to criminal motivation, as approximately 45% of psychopaths report being motivated by material gain [55].

Along the same line, Boccardi et al. [56] published a study describing the anatomical differences of the basal nuclei between healthy controls and psychopaths, as well as between subjects with varying degrees of psychopathy. The nucleus accumbens is a key structure in reversal learning, defined as the ability to modify previously acquired associations as context or task contingencies change. Successful reversal learning requires integrating experience, updating the value assigned to stimuli and goals, and predicting the consequences of actions. In other words, a behavior that was adaptive or rewarding in one context may lose its value in a different situation, and the individual must adjust based on prior knowledge to respond effectively. Given that psychopathic individuals show reduced sensitivity to punishment, impaired learning from negative outcomes, and a general reluctance to adjust behavior in corrective contexts, the nucleus accumbens becomes particularly relevant for investigating the neurobiological relationship between cognitive flexibility, volitional control, and psychopathic behavior. In this study, the authors found a symmetrical reduction of approximately 13% in the global volume of the nucleus accumbens, most pronounced in its rostral portion. Alongside this reduction, small hypertrophic areas were observed, leading to a more rounded morphology, which is atypical when compared with healthy controls. While these findings are consistent with similar previous research, the authors emphasize the need for more detailed analyses of individual limbic components rather than treating the limbic system as a unitary structure. One year later, Cope et al. [30] reported that individuals who had committed homicide showed significantly reduced bilateral gray matter volume in the caudate nucleus.

In 2022, Choy et al. [57] delved into the approach initially proposed by Raine and Glenn in 2010 [55], who brought attention to the inconsistency across studies reporting increased striatal volume in individuals with psychopathy. These discrepancies may reflect the difficulty in determining whether such enlargement is truly linked to psychopathy or instead to other clinical conditions or environmental influences. It was also emphasized the importance of studying women with psychopathy, noting that assuming neuroanatomical findings can be extrapolated to woman is inaccurate. Using MRI, the authors quantified striatal gray matter volume of 108 men and assessed psychopathic traits with the Hare Psychopathy Checklist. The same methodology was applied to a group of 12 women. To minimize confounding variables that have limited past studies, they controlled for ASPD, current or past substance use, Attention Deficit/Hyperactivity Disorder (ADHD), history of head trauma, total brain volume, age, ethnicity, and exposure to childhood abuse. Correlational analyses showed that larger striatal volume, specifically in the caudate nucleus, putamen, nucleus accumbens, and globus pallidus, was associated with higher psychopathic traits in both men and women. Compared with controls, psychopathic men showed a 9.4% increase in striatal gray matter volume, predominantly in the caudate, putamen, and nucleus accumbens. These findings support the hypothesis that enlargement of the striatum may contribute to an impulsive, reward-driven behavioral profile defined by increased sensitivity to immediate gratification, eventually transforming into a psychopathic trait. The authors conclude that further research is necessary to determine the origin of this volumetric increase.

Cavum Septum Pellucidum

The septum pellucidum is a deep limbic structure located in the midline, composed of two translucent sheets of glia that separate the lateral ventricles, forming part of the septohippocampal system. It is composed predominantly of ependymal glia and fiber tracts that run below the rostral portion and knee of the corpus callosum in the medial region of the frontal lobe [58].

At about 12 weeks of gestation, a small cavity forms between the two septal laminae, which is known as the cavum septum pellucidum (CSP). The closure of this space begins at the 20th week of gestation and is completed between 3 and 6 months after birth, which is attributed to rapid development of the alveoli of the hippocampus, amygdala, septal nuclei, fornix, and corpus callosum. Failure of this normal neurodevelopmental process prevents the fusion of the laminae, resulting in persistence of the CSP into adulthood. In humans, it has been proposed that abnormal development of the septal region may contribute to psychopathic, antisocial, and disinhibited behavior, given that disruptions in the septal nuclei could alter connectivity between limbic structures. This pathological behavior is hypothesized to manifest itself through an impaired attachment and a lack of prosocial affiliative behavior [58].

Raine et al. [58] proposed that abnormal neurodevelopment, reflected in the persistence of the CSP, may contribute to conditions such as ASPD and psychopathy. The authors studied a sample of 87 participants, comprising 75 men and 12 women. ASPD was diagnosed using DSM-IV criteria and the SCID-II, identifying 17 men and one woman with this disorder. Psychopathic traits were assessed with the Hare Psychopathy Checklist, considering scores ≥23 as indicative of significant psychopathy, grouping 30 men and two women. Criminal history was also recorded, revealing that 33 men and four women had arrest records, and 26 men and three women had court convictions. After classifying participants according to these clinical and behavioral variables, MRI scans were performed to assess the presence of CSP. The results concluded that individuals with CSP, particularly those diagnosed with ASPD with a predominance of the aggressive factor, displayed higher psychopathy scores and a greater number of criminal records compared to those without CSP. Regarding gender, men with CSP showed a higher prevalence of ASPD than women. Even participants without an ASPD diagnosis displayed a greater criminal history if they had persistent CSP, compared to individuals who did not have ASPD and had no criminal history. These findings suggest that aberrant limbic-septal neurodevelopment may contribute to a spectrum of maladaptive behaviors, ranging from increased risk of criminal conduct to traits associated with antisocial personality and psychopathy. The importance of this study lies in the notion that, if an anatomical structure reflects an abnormal embryonic process, preventive or early-intervention measures could be implemented during prenatal care and even throughout the first years of childhood, potentially improving long-term outcomes.

It is essential to emphasize that embryonic anatomical anomalies constitute only one of many factors influencing pathological behavior. Numerous studies highlight the detrimental role of environmental factors on postnatal neurodevelopment, including exposure to alcohol, nicotine, and malnutrition [58].

Cerebellum

The cerebellum is an oval-shaped organ, narrower in its middle portion, located in the posterior cranial fossa and covered superiorly by the tentorium cerebelli. It is composed of two cerebellar hemispheres joined by the vermis and is divided into three main lobes: the anterior, the posterior (also called the middle lobe), and the flocculonodular lobe. The anterior lobe is visible on the superior surface of the cerebellum and is separated from the posterior lobe by the primary fissure. The posterior lobe is the largest of the cerebellar lobes, and it extends between the primary and the posterolateral fissures. The flocculonodular lobe lies anterior to the posterolateral fissure [20].

The cerebellum has been traditionally associated with motor control, coordination, and balance; however, evidence suggests that specific regions are also involved in cognitive and learning processes [16].

Initial research focused on patients with congenital malformations, focal lesions, or localized neuronal degeneration. In 1998, Schmahmann et al. [59] explored the anatomical, physiological, and functional evidence supporting the involvement of the cerebellum in higher mental functions. Through multiple neurological and neuropsychological evaluations, as well as MRI scans in 20 patients with cerebellar pathology, they identified behavior-related symptoms, including impairments in executive functions (decision-making, working memory, set-shifting, verbal fluency, abstract reasoning), visuospatial deficits, emotional disturbances (obsessive-compulsive symptoms, disinhibition, flat affect), and linguistic alterations (agrammatism, dysprosody). They also found a stronger association of these symptoms in patients with involvement of the posterior cerebellar lobe and the vermis. The article concludes by designating this constellation of symptoms as the “Cognitive-Affective Cerebellar Syndrome.”

In 2007, the same authors published "The Neuropsychiatry of the Cerebellum - Insights From the Clinic," a case series describing various neuropsychiatric conditions resulting from congenital cerebellar abnormalities such as agenesis, dysplasia, and hypoplasia, as well as acquired conditions including cerebellar infarction, tumors, cerebellitis, trauma, and neurodegenerative diseases. Regarding aggressiveness and impaired behavioral control, the authors reported a case of cerebellar dysplasia in an 18-year-old patient, as well as a case of cerebellar histiocytosis diagnosed at three years of age and reassessed in young adulthood. They conclude by supporting the theory that the cerebellum plays an important role in attentional control, emotional regulation, autism spectrum disorders, psychotic disorders, and social cognition, with both positive and negative symptoms reflecting this involvement [60].

From a neurodevelopmental perspective, in that same year Giedd et al. [61] analyzed the impact of genetic and environmental factors on brain development during childhood and adolescence. They found that the cerebellum appears to be the brain structure with the lowest degree of heritability, and that environmental influences such as alcohol exposure, lead toxicity, and anoxia play a particularly significant role in its development.

Along the same lines, Bauer et al. [15] analyzed the effects of early separation from the primary family unit on cerebellar maturation and cognitive development. Their main hypothesis was that children institutionalized from birth would show developmental delays compared with children raised in stable family environments, and that they would also exhibit reduced cerebellar volume. Bauer begins by citing Rodolfo Llinás’ 1969 work "Neurobiology of Cerebellar Evolution and Development," in which he notes that cerebellar neurogenesis continues through the first two postnatal years and may undergo additional neuronal remodeling into late childhood and early adulthood. This motivated the authors to investigate how early postnatal experiences influence cerebellar development and its behavioral correlates. The study included 31 children (15 boys and 16 girls) who experienced early institutionalization from birth and multiple subsequent changes in caregiving environments. Most spent their early childhood in orphanages or state-run childcare institutions before later being adopted into stable family settings. There was evidence that these children exhibited motor, cognitive, and emotional difficulties, even years after adoption. The institutionalized group was compared with 30 children (16 boys and 14 girls) raised in family environments since birth. Both groups underwent MRI scans and seven standardized psychometric assessments evaluating multiple cognitive domains, described by the authors as part of the “adopted child complex”. The results showed that previously institutionalized children had reduced gray matter volume in the anterior and posterior cerebellar lobes, predominantly in the left hemisphere, and that they performed more poorly on cognitive measures than controls. Although the exact conditions of pre-adoption environments could not be fully reconstructed, the findings suggest that early environmental deprivation significantly influences cerebellar plasticity, with enduring consequences for cognitive and behavioral development.

Regarding research conducted during adolescence, Elmer and Cope [50] investigated the relationship between brain structure and psychopathic traits in incarcerated male youths housed in a maximum-security facility. Using MRI and the Hare Psychopathy Checklist in its adolescent version, they reported that individuals with elevated psychopathic traits showed a reduction in gray matter volume across extensive regions of the cerebellum. This decrease was interpreted as being consistent with a delay in neurodevelopment, although alternative explanations were also considered. A year later, the same authors published "Abnormal Brain Structure in Youth Who Commit Homicide," following a similar methodological model, except this time focusing on juvenile offenders who had committed homicide. The findings were concordant with the earlier study, revealing a significant reduction in gray matter volume in both cerebellar hemispheres [30].

Finally, in 2019, Sajous-Turner et al. [13] compared gray matter volume across three groups of adult violent offenders and reported that those who had committed homicide exhibited significantly lower cerebellar gray matter volume than both the violent but non-homicidal group and the minimally violent group.

Table 2 presents a summary of brain regions implicated in violent behavior.

**Table 2:** Brain regions implicated in violent behavior. This table summarizes key brain regions associated with violent or antisocial behavior, including their typical functional roles and findings from neuroimaging and neuropathological studies of violent individuals.

Brain region	Associated functions	Findings on violent behavior
Prefrontal cortex (PFC)	Impulse control, moral reasoning, decision-making [21,36]	Hypoactivity and volume reduction linked to antisocial and psychopathic traits [11,13,14,16,17,19,20,21,22,23,24,25,26,30,44,55].
Orbitofrontal cortex (OFC)	Evaluation of reward and punishment, as well as emotional and social behavior. Suppression of violent and aggressive feelings [21,36]	Antisocial behavior, aggression, and lack of remorse [11,13,14,16,19,20,21,22,23,24,25,26,27,28,30,44,45,47,50,56].
Ventromedial prefrontal cortex (vmPFC)	Affect regulation, reinforcement learning, caregiving behavior, and the processing of social norms and cultural values [19,20,27,33,55]	Gray and white matter volume changes have been observed in individuals with conduct disorder, executive function deficits, impaired social information processing, and response [11,13,14,19,20,24,27,34,50,55,56].
Dorsolateral prefrontal cortex (dlPFC)	Impulse management and mental functions, including risk-benefit assessments, problem-solving, and cognitive control [20]	Functional and anatomical abnormalities have been associated with the development of impulsive behavior, deficits in aggression control, and altered reward processing [20]. Reduced gray matter volume could lead to compromised impartiality and a tendency to act unfairly, which contributes to antisocial and aggressive behaviors [13,35].
Superior parietal lobe (SPL)	Multisensory and visuomotor integration, maintaining reciprocal connections with the PFC [36]	Reduced gray matter volume [13]. Reduced brain activity in individuals who had committed homicide [24,29,37].
Temporal lobe	Evaluating and integrating information related to social interactions [13]. Memory retention and storage, the organization of sensory stimulus input, language production, visual perception, and emotional response [19]	Bilateral reduction in gray matter volume [13,19,30] and abnormal electrical activity [19].
Occipital lobe	Reception, processing, recognition, and interpretation of visual information [36]	Gray matter volume reduction in ASPD-BPD and ASPD-PP [24].
Anterior cingulate cortex (ACC)	Cognition, error and conflict monitoring, emotion regulation, empathy, mental state attribution [21,36,38]	Volume changes that result in impaired social conduct, poor decision-making, and deficits in emotional processing. Impairments in attention, reduced cognitive flexibility, impulse-control deficits, and alterations in higher-order cognitive and self-regulatory processes [11,12,13,14,19,20,21,23,24,25,26,30,44,47,50,56].
Insula	Homeostasis, consciousness, self-awareness, empathy, interpersonal experiences, emotional processing, regulation of social behavior, integration and processing of conflicting information, and new learning [21,36]	Gray and white matter volume changes, altered connectivity in psychopathy [13,16,20,21,23,25,27,28,29,30,44,50].
Amygdala	Fear processing, emotional reactivity, linking memory to emotional events [21,36]	Hyperactivity in reactive aggression; hypoactivity in callous-unemotional traits. Gray matter volume changes according to age. Altered social perception [12,14,16,18,19,20,21,22,23,24,25,27,30,44,45,47,50,55,56].
Hippocampus	Encoding of new memories and emotional context, involvement in delayed fear responses [21,36]	Volume changes in violent offenders, as well as implications in negative affect and emotion regulation [12,16,19,20,21,23,27,28,30,44,45,50].
Thalamus and hypothalamus	The thalamus comprises 20 nuclei, functioning as a relay center, directing and exchanging sensory information between the different brain areas [19]. The hypothalamus regulates the autonomic nervous and endocrine systems, undertaking appropriate control responses by integrating the nervous and chemical data it receives [36]	Improvement in emotion after an ANT infarction is a paradoxical outcome [51]. Engagement of prefrontal thalamocortical circuitry in aggression following DBS [52]. Mediation of reactive aggression by the medial hypothalamus, as well as a direct relationship between physical aggression and hypothalamic activation [43].
Caudate nucleus	Action selection, motor and cognitive control, processing sensory stimuli, and predicting reward outcomes, using memory to support learning by drawing on past experiences [19,21,36]	Structural and functional changes related to impulsive behavior [28,29,44,55,57].
Striatum	It receives afferent information from most of the cerebral cortex, thalamus, subthalamus, and brainstem, including the substantia nigra [36]. Involvement in reward processing [42]	Activation of the dorsal striatum during punishment administration [42]. Total striatal volume increase and abnormal striatal activity in psychopathic individuals [55,57].
Nucleus accumbens	Reward, motivational, and fear processing. Reinforcement learning, impulsivity, reward-seeking behavior, and behavioral regulation [21,36]	Volume changes and hyperactivity in aggressive individuals [14,21,44,56,57].
Cavum septum pellucidum (CSP)	At about 12 weeks of gestation, a small cavity forms between the two septal laminae, which is known as the cavum septum pellucidum (CSP). The closure of this space begins at the 20th week of gestation and is completed between three and six months after birth [58].	Aberrant limbic-septal neurodevelopment may contribute to a spectrum of maladaptive behaviors, ranging from increased risk of criminal conduct to traits associated with antisocial personality and psychopathy [58].
Cerebellum	Motor control, coordination, and balance. Specific regions are also involved in cognitive and learning processes [16].	Lower gray matter volume in adolescents and adults [13,30,50]. Early environmental deprivation has a significant influence on cerebellar plasticity, with enduring consequences for cognitive and behavioral development [15]. Impairments in executive functions, visuospatial deficits, emotional disturbances, and linguistic alterations in patients with congenital malformations, focal lesions, or localized neuronal degeneration [59,60].

White matter correlates

Although most studies examining brain volume in aggressive or psychopathic individuals have concentrated on gray matter, the contribution of white matter should not be overlooked.

In 2003, Raine et al. [62] proposed five main objectives: to determine whether psychopathic individuals with ASPD exhibit structural and/or functional abnormalities of the corpus callosum; to assess whether these alterations are proportionally associated with functional impairments; to examine whether such findings parallel dimensional analyses conducted in a larger, non-selected sample; to investigate whether these abnormalities relate to core psychopathic traits, such as reduced emotional intelligence and difficulties sustaining interpersonal relationships; and, finally, to evaluate whether structural defects of the corpus callosum are independent of established risk factors for the development of antisocial behavior. The corpus callosum is the principal commissural fiber tract in the human brain and plays a key role in interhemispheric communication, emotional processing, and arousal regulation. Alterations in this structure can have significant consequences not only for cognition, affect, and emotion regulation, but also for psychopathologic status. In this case-control study, 15 men with ASPD and high scores on the Hare Psychopathy Checklist were compared with 25 healthy controls. The findings indicated that individuals with ASPD and psychopathy exhibited a 22.6% increase in callosal white matter volume, a 6.9% increase in overall length, a 15.3% reduction in average thickness, and enhanced interhemispheric functional connectivity relative to controls. Correlational analyses in the larger, non-selected sample supported the association between antisocial personality traits and structural abnormalities of the corpus callosum. The volumetric enlargement was linked to interpersonal and affective deficits (Factor 1 of the Hare Psychopathy Checklist), reduced autonomic reactivity to stress, and poorer spatial orientation. Moreover, the structural alterations appeared to be independent of established risk factors for the development of antisocial behavior. These findings suggest a neurodevelopmental deviation in this population, which may result in early axonal pruning or excessive myelination of callosal fibers.

Years later, Hoppenbrouwers et al. [15] examined white matter architecture to determine whether psychopathic individuals show abnormalities in large-scale neural circuits. A group of 11 psychopathic individuals was compared with 11 healthy controls using diffusion tensor imaging (DTI). A tract-based spatial statistics (TBSS) approach was employed to conduct a voxel-wise analysis of diffusion metrics across the whole brain, allowing data-driven identification of white matter alterations without a priori assumptions about which pathways might be compromised. The authors hypothesized disruptions in the tracts connecting the amygdala and nucleus accumbens with the PFC. TBSS analysis revealed five spatially non-contiguous clusters of reduced fractional anisotropy (FA) in psychopathic individuals. These clusters encompassed the bilateral uncinate fasciculus and the inferior fronto-occipital fasciculus, extending into the anterior subgenual cingulate, the left amygdala, the left OFC and the left frontal pole. Additional reductions in FA were observed in regions corresponding to anterior thalamic radiations bilaterally and their medial projections toward the anterior subgenual cingulate. Overall, this FA decreases were most pronounced in the left hemisphere. The three most affected clusters mapped onto two major neural systems: the amygdala-prefrontal network and the striatal-thalamic-frontal network.

**Figure 4:**
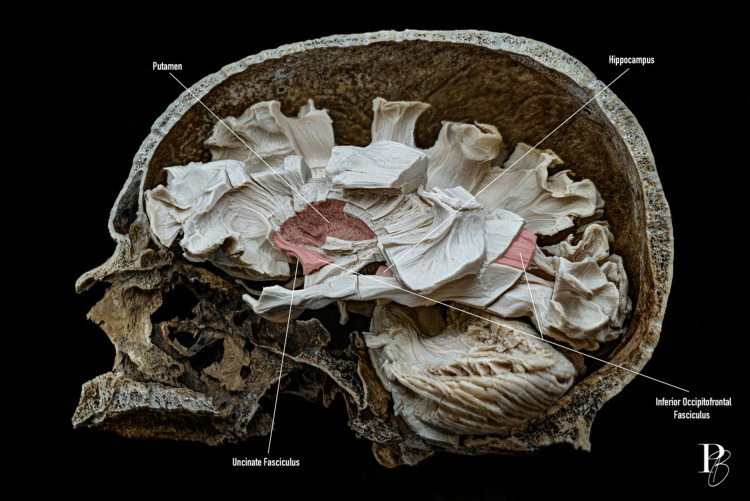
Sagittal dissection of white matter tracts and deep brain nuclei. Coronal dissection of the human brain, highlighting white matter tracts and deep grey matter structures. The putamen and hippocampus are shown in red. Two major white matter tracts are illustrated: the uncinate fasciculus, connecting the anterior temporal lobe with the orbitofrontal cortex, and the inferior occipitofrontal fasciculus, linking occipital and frontal regions. The photograph was taken by Dr. Daniel Ballesteros-Herrera, co-author of the work.

Figure 4 shows a sagittal dissection of white matter tracts and deep brain nuclei.

Regarding the association between these tractographic alterations and participants’ scores on the Hare Psychopathy Checklist, it was found that Factor 1, or the interpersonal/affective component, was linked to reduced white-matter integrity in the OFC and the frontal pole. In contrast, Factor 2, or the lifestyle/antisocial component, was associated with abnormalities within the striatal-thalamic-frontal network. The authors conclude by emphasizing that determining the origin of these white matter disruptions, whether they reflect atypical neurodevelopment or arise from environmental factors, remains a meaningful area for further investigation [15].

Finally, Pujol et al. [12] reviewed studies reporting white-matter abnormalities assessed through DTI metrics, particularly FA. Alterations in the ventral frontotemporal connections are one of the most consistent findings, primarily involving the uncinate fasciculus. A significant reduction in FA has also been observed in dorsal frontoparietal pathways. Notably, cingulate pathways appear altered both ventrally and dorsally, with decreased FA in the segment linking the PCC to the medial temporal lobe and in the segment connecting the PCC to the frontal lobe.

This reduction in FA has been interpreted as reflecting delayed maturation of major white-matter bundles; however, an accelerated developmental trajectory may better account for the patterns observed in psychopathic individuals. Typically, FA peaks between 20 and 40 years of age and subsequently declines, indicating that its normative course follows a gradual post-peak reduction. In most DTI studies, participants are within the age range corresponding to peak FA or the initial phase of its decline; therefore, the frequently reported reductions could be more consistent with premature maturation. Findings from DTI studies in younger individuals with callous-unemotional traits have been mixed, showing significant reductions, minimal differences, or no group differences in FA. Collectively, these results support the hypothesis that an atypical acceleration in white-matter maturation contributes to the structural connectivity alterations observed in psychopathy and callous-unemotional traits [12].

Discussion

Table 3 presents the theoretical models proposing links between brain structure and violent behavior.

**Table 3:** Diagram of theoretical models linking brain structure to violent behavior. This table summarizes key neurobiological models from the manuscript that explain the relationship between specific brain alterations and violent or psychopathic behavior. Each model lists its main proponents, central hypothesis, and implicated neural correlates. The data are drawn exclusively from studies cited within this review, without reference to external sources. PFC, prefrontal cortex; OFC, orbitofrontal cortex; vmPFC, ventromedial prefrontal cortex; ACC, anterior cingulate cortex

Model/Theory	Key proponents	Core idea	Neural correlates
Dark Core of Personality	Moshagen et al. [5]	A latent “D factor” unifies dark traits like psychopathy, narcissism, and sadism, predisposing to violent behavior.	Reduced gray matter in the PFC, amygdala, and insula.
Dynamic Gray Matter Evolution	Pujol et al. [12]	Gray matter volume in individuals with psychopathic traits changes with age, increasing in childhood and decreasing in adolescence.	Gray matter increases in childhood, then decreases during adolescence in the PFC and temporal lobes.
Frontal Lobe Dysfunction	Hornak et al. [39]; Yang and Raine [40]	Damage to prefrontal areas (especially OFC and vmPFC) impairs impulse control, empathy, and moral judgment.	OFC, vmPFC, ventral ACC
Cavum Septum Pellucidum Persistence	Raine et al. [58]	Persistence of the cavum septum pellucidum into adulthood correlates with a higher risk of antisocial behavior and criminality.	Limbic system: septal nuclei, hippocampus, amygdala
Accelerated White Matter Maturation	Pujol et al. [12]	Psychopathic traits may be linked to early maturation of white matter bundles, especially in frontotemporal circuits.	White matter: uncinate fasciculus, corpus callosum, frontotemporal connections

The findings of this review provide important neuroanatomical insights for both neuroscientific research and the prevention of violent behavior. However, although neuroscience has enabled significant advances in the understanding of antisocial and homicidal behavior, these findings should not be interpreted as deterministic. Environmental factors such as childhood abuse, social deprivation, and exposure to violence can interact with biological predispositions, shaping the development of criminal behavior.

Table 4 presents neuroimaging techniques and their corresponding findings in studies of violent behavior.

**Table 4:** Neuroimaging techniques and findings in studies on violence. This table compares the primary neuroimaging modalities used to study violent behavior, highlighting each technique’s focus and the most consistent findings in individuals exhibiting such behavior. PFC, prefrontal cortex; vmPFC, ventromedial prefrontal cortex; dmPFC, dorsomedial prefrontal cortex; vlPFC, ventrolateral prefrontal cortex; dlPFC, dorsolateral prefrontal cortex; OFC, orbitofrontal cortex; PCC, posterior cingulate cortex; mOFC, medial orbitofrontal cortex; ACC, anterior cingulate cortex; CSP, cavum septum pellucidum

Imaging technique	Focus of analysis	Common findings in violent subjects
Structural magnetic resonance imaging (MRI)	Gray matter volume and cortical thickness. Corpus callosum structural abnormalities.	Reduced volume in PFC, amygdala, hippocampus, insula [11,13,16,18,30,47,50,56], vmPFC [11,13,24], dmPFC [13,24], vlPFC [13], dlPFC [13], postcentral gyri, frontopolar cortex, OFC [11,13,16,24,25,28,29,50], PCC [11,13,24,50], bilateral temporal lobes [11,13,17,27,30,50], nucleus accumbens [29], superior parietal regions [13], occipital cortex [24], and superior-posterior cerebellar lobes [13,15,16,17]. Increased striatum volumes [55,57]. Increased gray matter volume in the mOFC and ACC, as well as bilateral temporal lobes in 11-year-old boys [23]. Persistence of CSP [58]. Increased estimated callosal white matter volume, increased callosal length, and reduced callosal thickness [62]. Increased bilateral white matter volumes in the occipital and parietal lobes, and in the left cerebellum [28].
Functional magnetic resonance imaging (fMRI)	Task-based or resting-state brain activity.	Hypoactivation in PFC during moral reasoning; hyperactivation during retaliation (PFC, ACC, anterior insula, right middle occipital lobe, right superior temporal gyrus, right inferior temporal gyrus, bilateral inferior parietal lobe, bilateral fusiform gyrus, right supramarginal gyrus, left hippocampus, right angular gyrus, amygdala) [22,44,45,54]. Hyperactivation during anticipation of pain (right superior temporal lobe, right postcentral gyrus, left middle temporal lobe, right pars triangularis of the inferior frontal gyrus, bilateral occipital lobe, bilateral inferior OFC, precentral gyrus, postcentral gyrus, pallidum, thalamus, supplementary motor area, and right somatosensory cortex) [45].
Diffusion Tensor Imaging (DTI)	White matter tract integrity.	Altered connectivity between the limbic system and prefrontal control regions [14].

The identification of neuroanatomical patterns associated with psychopathy and aggressiveness suggests the possibility of developing functional neuroimaging tools to detect individuals at risk for antisocial behavior at an early stage. Such tools could support the implementation of early intervention strategies aimed at addressing emotional dysregulation and strengthening prosocial skill development in vulnerable populations. Future research should prioritize longitudinal and multidisciplinary approaches that allow a more precise establishment of the causal relationships between brain structure and antisocial behavior. Likewise, the integration of neuroimaging, genetics, and computational models may improve the identification of individuals with a predisposition toward violence and open new avenues for rehabilitation. Ultimately, these advances raise significant ethical and legal concerns, particularly regarding criminal responsibility and the use of neuroscience in forensic settings. Therefore, an interdisciplinary debate on their applications is essential.

## Conclusions

The evidence analyzed suggests consistent neuroanatomical patterns in individuals with violent or psychopathic behavior, characterized by alterations in gray matter volume in key regions such as the PFC, temporal lobe, cingulate cortex, and insula, as well as changes in white matter organization and interhemispheric connectivity. These findings support the hypothesis that, from a neurobiological perspective, evil is not merely a social construct but may be associated with structural and functional differences in the brain.
